# Improvement in MR quality control workflow and outcomes with a web‐based database

**DOI:** 10.1002/acm2.12879

**Published:** 2020-04-19

**Authors:** Xiangyu Yang, Kevin Little, Xia Jiang, David Hintenlang

**Affiliations:** ^1^ Department of Radiology The Ohio State University College of Medicine Columbus OH USA

**Keywords:** automated quality control, human error, MRI, web‐based database

## Abstract

**Purpose:**

To describe a custom‐built, web‐based MR Quality Control (QC) database, and to assess its impact on the QC workflow and outcomes in a large U.S. academic medical center.

**Methods:**

The MR QC database was built with Microsoft Access 2010 and published on a Microsoft Sharepoint website owned and maintained by the authors' institution. Authorized users can access the database remotely with mainstream web browsers on any institutional computers. QC technologists were granted access to add, review, and print daily and weekly QC records. Qualified medical physicists (QMPs) were granted additional access to edit, review, and approve existing QC records and to change tolerance limits. A macro was utilized to conduct an automatic weekly review of QC status and to email the results to a QMP. This web‐based QC database was implemented on 17 clinical MRIs at the authors' institution. Weekly ACR QC findings within one year before and after implementation were compared.

**Results:**

We analyzed 158 QC issues detected by the web‐based database and 127 QC issues identified in conventional paper records before we implemented the database. The web‐based database significantly reduced the number of QC issues due to technologist error (before/after: 59/24 cases, *P* < 0.0001) but did not affect the number of QC issues related to scanner performance (before/after: 49/46 cases, *P* = 1). Further analysis revealed that the web‐based database significantly reduced the average time for the QMPs to identify a QC issue (before/after: 177 ± 110/2 ± 2 days, *P* < 0.0001) and time to correction (before/after: 81 ± 102/7 ± 8 days, *P* < 0.0001). The correction rate also significantly increased (before/after: 22%/99%, *P* < 0.0001).

**Conclusion:**

The web‐based QC database provides a positive impact on our MR QC workflow and outcomes. It simplifies QC workflow, enables early detection of quality issues, and facilitates quick resolution of problems that may affect the quality of clinical MRI studies.

## 1 INTRODUCTION

Quality control (QC) is an essential component of radiologic practice. A well‐designed and well‐executed QC program allows for imaging service providers to identify problems in the early stage of their manifestation and to take proper corrective actions with minimal interruption to clinical service. In Europe and North America, regulatory agencies and accrediting bodies have imposed rigorous requirements on QC programs of imaging modalities under their governance. In the United States, the American College of Radiology (ACR) developed dedicated phantoms and QC procedures for various imaging modalities.^1^, ^2^, ^3^ These procedures and phantoms are being used routinely at more than 38,000 ACR‐accredited imaging facilities in practices of Mammography, Computed Tomography (CT), Magnetic Resonance Imaging (MRI), and Nuclear Medicine (NM).

An effective QC program requires coherent and persistent efforts by radiologists, technologists, and qualified medical physicists (QMPs). Specially trained QC technologists are the first‐line workers in radiologic QC programs. Under the technical oversight of QMPs and the supervision of radiologists, they play a vital role in maintaining high quality performance of imaging equipment. They conduct basic QC testing on a daily or weekly basis, report quality issues to the supervising QMPs and radiologists, and initiate corrective actions following QMP's instructions. This mechanism, however, could be substantially compromised by human error or negligence. Scheduled QC tests could be neglected due to miscommunication or an unexpected increase in clinical workload. QC technologists could make mistakes in the evaluation of QC data or inadvertently overlook a test result outside the tolerance limits. Periodic QMP review of QC logs provides an opportunity to identify and address these issues, but it is usually difficult to conduct the QMP review at sufficiently high frequency. Consequently, quality issues could persist and accumulate in the clinical operation of radiologic imaging services for a prolonged period before they are identified, potentially leading to decreased patient care quality or safety and citations by regulatory agencies or accrediting bodies.

We hypothesize that the limitations of a traditional paper‐based QC record discussed above can be overcome with a web‐based QC system that allows for centralized management of multisite QC data, real‐time detection of QC issues, and automated reporting to a QMP. To the best of our knowledge, the impact of web‐based QC systems has not been systematically evaluated for clinical environments in scientific literature despite the presence of a few automated QC image analysis tools^4^, ^5^, ^6^, ^7^ and commercial enterprise solutions.^8^, ^9^ In this paper, we describe a custom‐built, web‐based MR QC database, report our experience with implementing such a system in a large academic medical center in the United States, and assess its impact on our QC workflow and outcomes.

## 2 METHODS

### 2.A The MR QC database

We built an MR QC web database with Microsoft Office Access 2010 and published it on a Microsoft Sharepoint 2010 website owned and maintained by the Information Technology Department at our institution. Authorized users can access the database with mainstream web browsers on any computer on our intranet. User access is controlled with a whitelist mechanism. There are two user groups with different privileges. All MR QC technologists are granted basic permissions to record, review, and print QC data. Advanced users (i.e., the QMPs) have full permission to amend or comment on an existing QC record, to set up new tolerance limits, and to manage the equipment list and user groups.

**Fig. 1:**
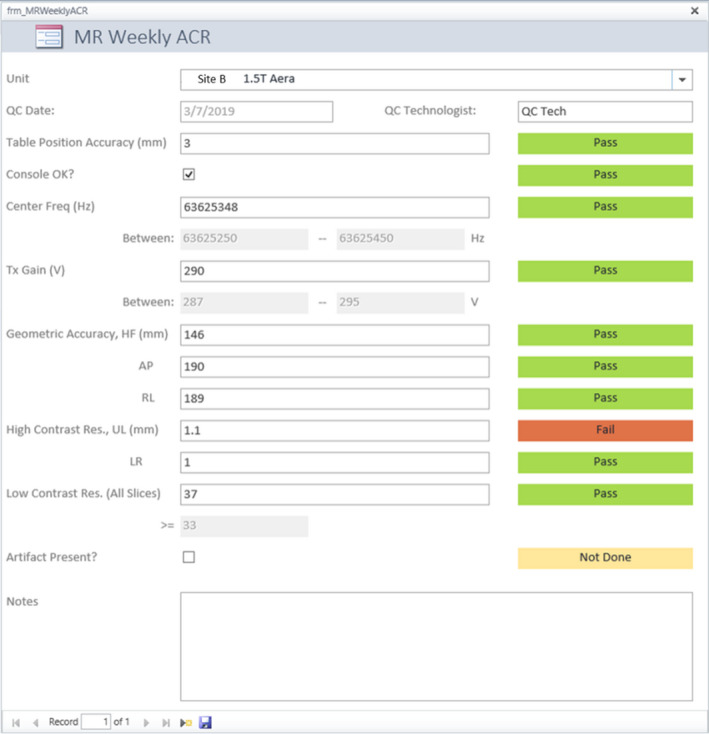
ACR weekly phantom data entry form. Specially trained QC technologists can add QC records to the MR QC database using Access web forms. This example is a web form for the collection of weekly ACR phantom data. Embedded macros were used to compare data entries with tolerance limits specified by a QMP. Passed, failed, and unfinished tests are respectively color coded as green, red, and yellow. A note can be added to the record to document QC technologist's comment, repeated testing results, or any other relevant information. ACR, American College of Radiology; QMPs, Qualified medical physicists.

**Fig. 2:**
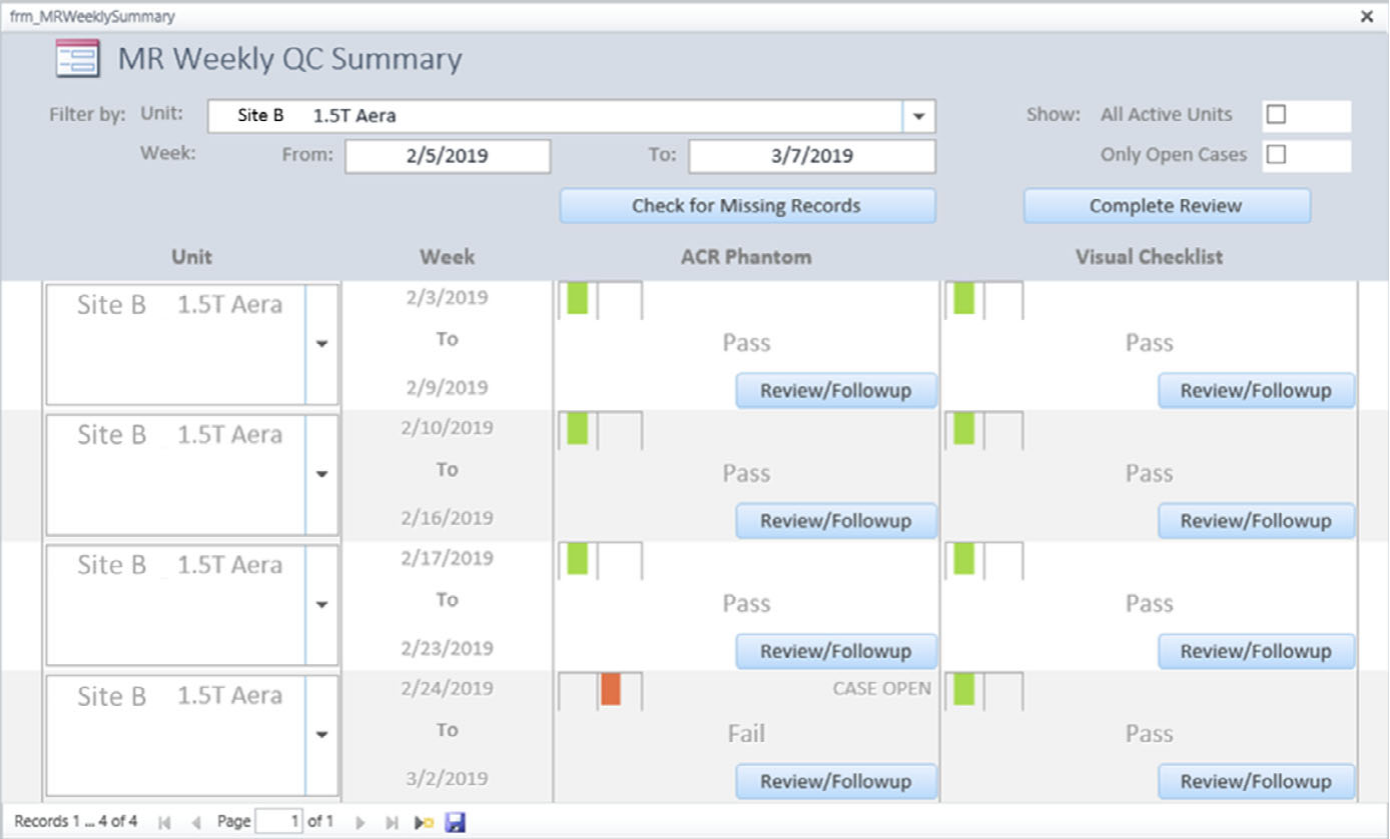
The QC Summary interface. This interface provides an overview of weekly QC status. QC records can be filtered by MRI unit or by time. Each missing or failed QC is labeled as an “open case” with a red case indicator. QMPs can track these cases with the “Review/Followup” function and amend their reports with assessment of the underlying problem or progress of corrective actions. Once an issue is resolved, the corresponding case(s) can be closed by a QMP. The case indicator is set back to green at the time of case closure. QMPs, Qualified medical physicists.

QC data are collected with web forms. Currently, this database accepts data from ACR weekly phantom tests and visual checklist, weekly Magnet Rundown Unit (MRU) test for General Electric (GE) MRIs, and manufacturer's daily QC for selected models of GE and Siemens scanners. The data entry forms use embedded macros to detect out‐of‐tolerance data fields in real time and label them with red color to alert the users (Fig. 1). At the end of each week, an Access macro scans all data entries in the previous week for missing records or QC failures. Each finding opens a case in the database, and the QMPs are notified by automatic email alerts. All active cases are displayed in chronological order on a QC Summary interface for QMPs' follow‐up until their resolution (Fig. 2). The QMP's assessment result, the course and outcome of corrective actions taken, and any other relevant information can be documented in a text note attached to the record.

### 2.B Implementation and Impact Assessment

In November and December of 2017, we deployed the MR QC database at our institution, a large U.S. academic medical center that serves a metropolitan area and surrounding suburban communities. The database covered 17 clinical MRIs distributed in 9 imaging facilities (Table 1). Operation of these scanners was overseen by a group of four QMPs certified by the American Board of Radiology (ABR) in diagnostic medical physics. Identical quality standards were applied to all types of facilities. All facilities have designated QC technologist(s), who were provided with a detailed user manual and in‐person training opportunities before the web‐based database was launched.

**Table 1:** Sites and MRI scanners involved in this study.

Site	Site type	MRI manufacturer	MRI model	Field strength (T)
A	General Hospital	GE	HDX	1.5
		Siemens	Espree^a^	1.5
		Siemens	Verio	3
B	Specialty Hospital	GE	MR450 (Rad Onc MR Simulator)	1.5
		GE	MR750 (Intraoperative)	3
		Siemens	Aera	1.5
		Siemens	Skyra	3
C	General Hospital	GE	Signa	1.5
D	Outpatient Clinic	Siemens	Avanto	1.5
		Siemens	Trio^b^	3
		Siemens	Verio	3
E	Outpatient Clinic	Siemens	Avanto (Mobile)	1.5
		Siemens	Skyra	3
F	Outpatient Clinic	Siemens	Verio	3
G	Outpatient Clinic	Siemens	Verio	3
H	Outpatient Clinic	GE	HDX	1.5
I	Outpatient Clinic	Siemens	Skyra	3

^a^ Replaced by a Siemens Aera 1.5T scanner in May, 2018

^b^ Upgraded to a Siemens Prisma 3T scanner in May, 2018.

In order to assess the impact of the web‐based QC approach, we collected and analyzed all weekly ACR QC findings detected by the database within one year (52 weeks) after its implementation. For comparison purposes, we also thoroughly reviewed all paper QC records from the year prior to the implementation to identify QC issues that occurred in that period. Daily QC data were not included in our analysis due to varied scopes of manufacturers' routine QC tests. A QMP reviewed all findings to determine their root causes. For issues requiring correction, the nature, timing, and extent of corrective actions were identified from relevant service reports, email communications, and interviews with the QC technologists involved.

During the study period, there were no personnel, procedural, or standard changes that could potentially confound our analysis. All contents of paper and electronic forms were kept identical. Two scanners were replaced by or upgraded to more advanced models after the database implementation. The total downtime due to scanner replacement and upgrade was 5 scanner‐weeks. In total, 884 scanner‐weeks of paper QC records and 879 scanner‐weeks of electronic QC records were included in our analysis. When multiple QC records were available within a single week due to baseline data collection after the new installation, only the last record was included in our analysis. The web‐based database underwent a service disruption for one week due to a major upgrade of the SharePoint service and consecutive database migration. All QC technologists were instructed to keep paper copies of their QC records and to add them to the database after the service was resumed. These expected delays were not counted as QC issues in our analysis. When a scanner failed multiple tests in the same week, they were counted as separate issues.

Statistical analysis was performed with Matlab (The MathWorks, Inc., Natick, MA). Comparisons were conducted on QC issues' occurrence frequencies, time to identification, and time to resolution before and after the implementation of the web‐based database. Frequencies were compared with Fisher's exact test. Continuous variables were compared with two‐sample Student's t‐test. When multiple comparisons were conducted simultaneously, the Bonferroni correction was applied to the P values.

## 3 RESULTS

**Fig. 3:**
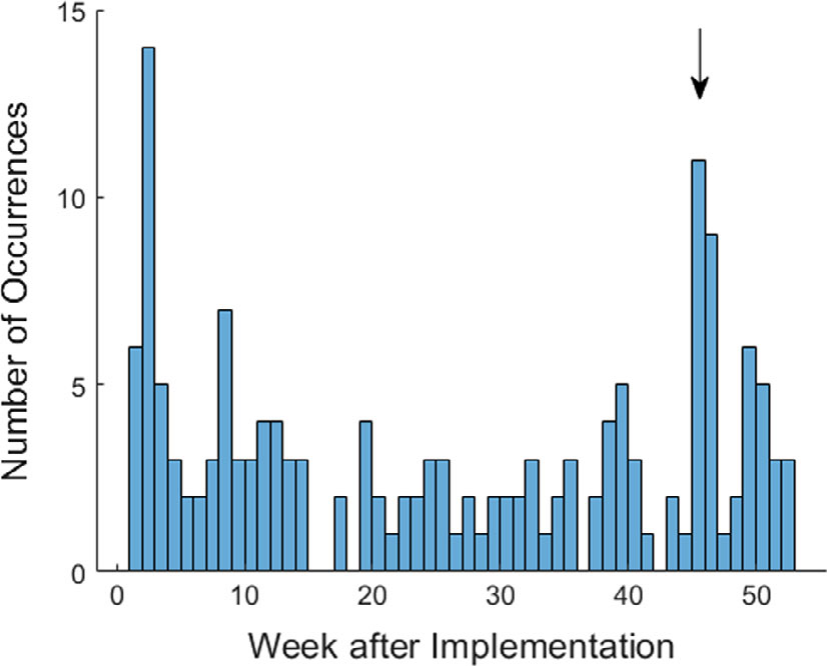
QC issue occurrence as a function of time after the implementation of the web‐based database. Occurrence of QC issues peaked in the transition period at week 2 when the QC technologists were still learning the new tool. But their performance improved quickly. By week 4, the occurrence had already dropped to a stable level. Another peak at weeks 45 and 46 (arrow) was due to a single event of miscommunication. A QC technologist in charge of three scanners at site A forgot to request database access for his backup before he left for vacation, leading to delayed logging of six QC records per week (three ACR phantom and three Visual Checklists) in these two weeks.

The MR QC database was smoothly implemented at all imaging facilities without any complaints from the technologists. The QC technologists mastered the new tool within a short time (Fig. 3). User feedback indicated improvements in QC workflow in several aspects: the QC technologists found it easier to handle changes in tolerance limits. The QMPs were able to review QC results in less time and at higher frequency. The case tracking function facilitated follow‐up of quality issues and enabled QMPs to close the loop after a proper corrective action was taken. The centralized data storage simplified data, equipment, and personnel management by QMPs and administrators.

We identified 127 QC issues in retrospective review of 884 scanner‐weeks of paper records. The web‐based database detected 158 QC issues in 879 scanner‐weeks of electronic records. QC technologists only reported a small fraction of these issues to the QMPs at the time of occurrence (5% prior to and 11% after the implementation of the web‐based database). Most issues were resolved within one or two weeks, but a few overlooked ones persisted for a long time. Prior to the implementation of the web‐based database, six QC issues had persisted for more than one month without being reported by QC technologists to the QMPs. One scanner repeatedly failed the transmitter gain test for more than six months until the problem was eventually discovered by a QMP at the subsequent annual evaluation. The web‐based database greatly enhanced the QMPs' ability to identify these overlooked or unreported quality problems. Its implementation led to a significant reduction in the average time to identify such an issue from 177 ± 110 days to 2 ± 2 days (*P* < 0.0001). Consequently, persistent QC issues due to operator negligence were eliminated.

Table 2 summarizes the breakdown of QC issues by their types. Missing records were by far the most frequently occurring QC problem in our practice. Their frequency of occurrence was 6% in paper records and 11% in the database. Other common QC issues include abrupt change in center frequency (4% in paper records, 4% in database), abnormal transmitter gain/attenuation (2% in paper record, 1% in database), inaccurate table position (1% in paper records, 1% in database), and geometric accuracy failures (1% in paper records, 0% in database). The majority (71/100) of missing records identified in the database were due to delayed evaluation or documentation of QC tests that were performed on schedule. The frequency of truly unperformed QC tests decreased significantly after we switched to the web‐based QC approach (from 44 cases, 5% to 19 cases, 2%; Bonferroni‐corrected *P* = 0.0209). There was also a significant decrease in the frequency of recorded geometric accuracy failures (from 10 cases, 1% to 0 cases, 0%; Bonferroni‐corrected *P* = 0.0209; see Discussions). Frequency changes in all other categories did not reach statistical significance. Overall, the web‐based database significantly reduced our QC failure rate (excluding delayed data loggings) from 14% (127 cases) to 10% (87 cases; Bonferroni‐corrected *P* = 0.0484).

**Table 2:** Breakdown of QC issues by type.

Type	Number (frequency) of occurrence		*P* value^b^
	Before	After	
Missing record due to delayed data logging	*Undetectable*	71 (8%)	*—*
Missing record due to scanner down^a^	5 (1%)	10 (1%)	1
Missing record due to neglected QC/lost data	44 (5%)	19 (2%)	0.0209^c^
Center frequency	34 (4%)	34 (4%)	1
Transmitter gain/attenuation	16 (2%)	9 (1%)	1
Table position	10 (1%)	7 (1%)	1
Geometric accuracy	10 (1%)	0 (0%)	0.0209^c^
Artifact	4 (1%)	3 (<1%)	1
Console	1 (<1%)	3 (<1%)	1
Low contrast	1 (<1%)	2 (<1%)	1
High contrast	2 (<1%)	0 (0%)	1
Total (without delayed data logging)	*127 (14%)*	*87 (10%)*	*0.0484* ^c^
Total	*127 (14%)*	*158 (18%)*	*—*

^a^ Downtime due to scanner upgrade/replacement were excluded.

^b^ With Bonferroni correction for multi‐comparison.

^c^ Statistically significant.

**Fig. 4:**
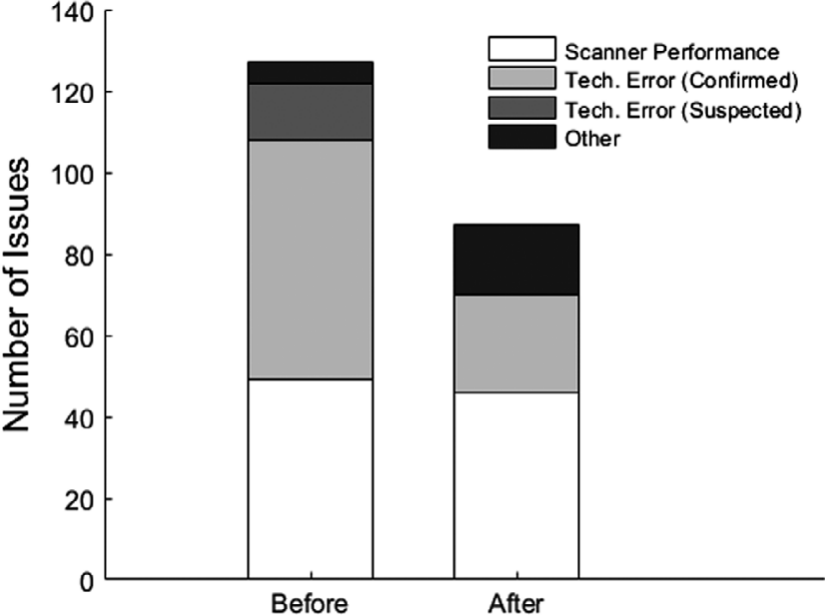
A breakdown of QC issues by cause. The “suspected technologist error” category includes QC failures (in geometric accuracy, high contrast resolution, and artifact) that are isolated events without any record of corrective actions leading to their resolution. The “other” category includes missing QC records due to unexpected scanner downtime or conflicts with busy clinical schedule.

Among the 127 QC issues identified in paper records, 59 cases (46%) were caused by technologist error (including neglected QC tests, incorrect execution of QC procedures, and transcription errors). Another 14 cases (11%) were suspected to be technologist error but could not be confirmed. After we started using the web‐based database, occurrence of technologist errors decreased significantly. Only 24 QC issues identified in the database (28%) could be attributed to technologist error (Bonferroni‐corrected *P* < 0.0001). The frequency of QC issues related to scanner performance remained virtually unchanged (49 cases in paper records, 46 cases in database; Bonferroni‐corrected *P* = 1; Fig. 4).

**Fig. 5:**
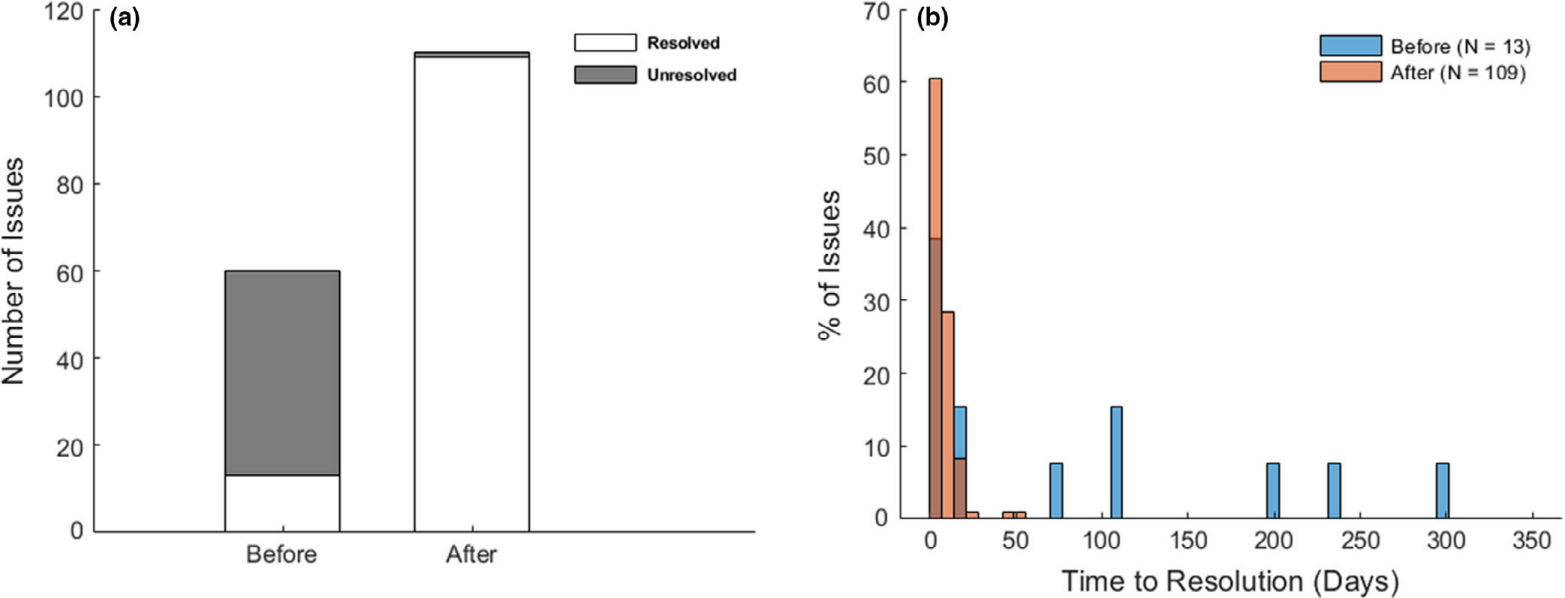
(a). The web‐based MR QC database substantially improved resolution of QC issues; (b). Distribution of resolution times before (blue) and after (orange) implementation of the web‐based database. The resolution time was significantly reduced after the implementation.

According to a QMP's assessment, corrective actions were deemed necessary for 60 (47%) QC issues occurring before the implementation and 110 (70%) issues occurring after the implementation. All but one of the issues detected by the database were addressed with proper corrective actions. The only exception occurred during the service disruption and database migration. Contrarily, the rate of correction was significantly lower prior to the implementation. Only 13 issues identified in the paper records were corrected (22%; *P* < 0.0001). The web‐based database also significantly reduced the average time to correction from 81 ± 102 days to 7 ± 8 days through timely detection and handling of QC issues overlooked by the technologist (*P* < 0.0001; Fig. 5).

The majority of corrective actions were taken in house. Only five cases before (8%) and nine cases after (7%) implementation required service correction. Services taken to address these issues include: laser calibration (three cases before and five cases after implementation), coil check/tuning (two cases after implementation), coil replacement (two cases after implementation, associated with the same coil and unrelated to the check/tunings mentioned above), and re‐engagement of table clutch (one case before implementation). No records of corrective action were found for one case occurring prior to the implementation. There seemed to be a trend of decreasing time to service resolution (from 51 ± 45 days to 20 ± 18 days), but the difference did not reach statistical significance, which is likely due to the small sample size (*P* = 0.09).

## 4 DISCUSSION

In radiologic QC programs, the QC technologists are expected to be able to effectively identify quality issues in their daily work and to efficiently relay them to supervisors or QMPs for further assessment and resolution. Our data, however, revealed that a nontrivial level of operator negligence and human error existed in the operation of a large institution despite its experienced imaging personnel, full access to physics expertise, and continuous promotion of a quality culture. The report rate was also found to be surprisingly low. These factors must be taken into account in the design, implementation, and execution of QC programs.

Even though quality issues requiring immediate service correction are expected to be rare in practice, negligence of such an issue could lead to serious consequences, including compromised patient care, increased safety risks to the patient and staff, and associated legal liabilities. While staff education is a useful way to cultivate a quality culture in an organization, it may be less effective in correcting inadvertent behaviors. Reducing the possibility of human error through process automation would be a more viable solution to this problem.

Our web‐based database addresses human error and negligence in several ways. Automatic evaluation of test results not only adds an additional check step in the QC workflow but also reduces the risk of applying wrong or outdated tolerance limits to the data. This is particularly important for QC tests with scanner‐specific or changeable tolerance limits, such as the transmitter gain or center frequency test. In our system, QC technologists receive real‐time feedback from color‐coded indicators on the data entry form. Our data suggested that these indicators are quite effective in alerting the QC technologists to the occurrence of QC issues and reminding technologists to take timely actions. For example, occurrence of geometric accuracy failures was high in our practice prior to the implementation of the web‐based database. All these failures, except for one case of an obvious transcription error, were isolated events where the results invariably returned to the normal values in the subsequent weeks without any service record of gradient calibration. Therefore, they were unlikely to be true indications of poor gradient performance, but rather indicated potential human errors in recording or interpreting the QC data. After we switched to the web‐based QC approach, the QC technologists can easily identify geometric accuracy failures with the help of color‐coded indicators and check the validity of their findings with a repeated scan. Consequently, the occurrence of geometric accuracy failures dropped to zero in the following year. This example provides an excellent demonstration of human error reduction through automated feedback to the technologists.

Automatic reporting of QC findings to a QMP adds another layer of fault prevention to the QC program. This function enables the QMPs to conduct frequent monitoring of QC data without substantially increasing their already heavy workload. Even if technologist error and negligence cannot be completely prevented, QMPs' oversight provides a mechanism for timely identification of overlooked quality issues and prompt initiation of corrective actions. The effectiveness of this mechanism has been proven by the significant decreases in response times observed in our study.

An interesting finding of our study was the significant decrease in neglected tests after the implementation of the web‐based database. This effect was rather unexpected because switching from paper record to the web‐based database only changed the way how QC data were collected, evaluated, and monitored. It should not have affected the process of generating those data. We speculate that two factors might have contributed to this effect: increased communication and less data loss. Interactions between QMPs and technologists increased after we implemented the web‐based database. QMPs' inquiry for information and instruction of corrective actions might have served as de facto reminders for the QC technologists, helping them better keep up with the QC schedule. We also noticed that delayed data logging beyond one weekly reporting cycle was quite common in our practice (occurrence frequency 8%). It was possible that miscommunication or negligence could have caused permanent loss of some undocumented data, thus yielding a higher rate of missing records. With an automatic weekly check of QC status, the web‐based database can detect delayed data logging within a few days after its occurrence, reducing the likelihood of data loss.

In house development of a web‐based QC system demands expertise beyond the scope of traditional radiologic practice. The QMPs need to have a working knowledge of database and network architecture to ensure proper integration of such a system into their workflow. After the implementation, the QMPs need to partner with Information Technology specialists to proactively discover and resolve potential technical problems. The imaging team needs to have an emergency response plan to handle unexpected service disruptions, and periodic backup of the database is strongly recommended. These requirements, however, should not be mistakenly regarded as barriers that should prevent small imaging facilities without access to these resources from reaping the benefits of web‐based QC. Contrarily, web‐based QC systems are particularly valuable to imaging facilities without an in‐house physicist because they give these users access to real‐time, high quality tele‐services provided by experienced QMPs, which could be difficult to obtain with conventional approaches.

We would like to stress that the web‐based QC approach is a supplement to, not a replacement of, other quality improvement efforts. Advertent errors such as the technologist's bias to get a passing result might be better addressed with staff education and culture improvement. The problem of delay and data loss might be mitigated with workload balancing and process optimization, since our data indicate that busy clinical schedule is a major cause of delays. In order to achieve excellence, continuous quality improvement must be made along all dimensions of imaging service.

Although our work only provides an example of a web‐based QC system for one imaging modality, the general principles and methods are applicable to other modalities as well. Since the mechanisms of improvement discussed in this paper are not specific to the MRI modality, we believe that other radiologic imaging modalities will similarly benefit from automation of their QC procedures.

Our study has several limitations. First, while our institution had nine distinct MRI facilities in the metropolitan area, it is effectively a single institution study. Our web‐based database solution was limited by the resources available at our institution at the time of its development. Other institutions may find alternative solutions, such as those that are based on Microsoft PowerApps or Oracle Database, which may be more suitable for their environment. Secondly, in this study we only focused on the improvement of QC workflow from QMPs' perspective. Our automated QC system could be further enhanced by integrating an automatic image analysis tool as described in the literature.^4^, ^5^, ^6^ Lastly, we only studied weekly QC tests required by the ACR. Although daily QC is not an ACR requirement, it is recommended by all major MRI manufacturers and is widely used in clinical facilities.^5^, ^10^, ^11^ It is aso a crucial component in our MR QC program. Our database has the capacity to integrate daily QC data from selected MRI models, but those data were not included in this study because different manufacturers' recommended tests vary significantly in their scopes. The web‐based QC approach's impact on daily QC needs to be assessed with future research.

## 5 CONCLUSION

Our study demonstrated the feasibility of developing a web‐based QC solution for a radiologic imaging modality and implementing it in a complex clinical environment. Our data clearly demonstrated that the web‐based database can simplify the QC workflow, reduce human errors, enable early detection of quality issues, and facilitate timely resolution of problems that may affect the quality of clinical MRI studies.

## CONFLICT OF INTEREST

The authors have no conflict of interest to disclose.

## References

[1] American College of Radiology . 2015 magnetic resonance imaging quality control manual. Reston, VA: American College of Radiology; 2015.

[2] American College of Radiology . 2017 computed tomography quality control manual. Reston, VA: American College of Radiology; 2017.

[3] American College of Radiology . 2018 digital mammography quality control manual. Reston, VA: American College of Radiology; 2018.

[4] Sun J , Barnes M , Dowling J , Menk F , Stanwell P , Greer PB . An open source automatic quality assurance (OSAQA) tool for the ACR MRI phantom. Australas Phys Eng Sci Med. 2015;38:39–46. 25412885 10.1007/s13246-014-0311-8

[5] Peltonen JI , Mäkelä T , Sofiev A , Salli E . An automatic image processing workflow for daily magnetic resonance imaging quality assurance. J Digit Imaging. 2017;30:163–171 27834027 10.1007/s10278-016-9919-4 PMC5359204

[6] Nowik P , Bujila R , Poludniowski G , Fransson A . Quality control of CT systems by automated monitoring of key performance indicators: a two‐year study. J Appl Clin Med Phys. 2015;16:254–265. 26219012 10.1120/jacmp.v16i4.5469 PMC5690007

[7] Lu W , Dong K , Cui D , Jiao Q , Qiu J . Quality assurance of human functional magnetic resonance imaging: a literature review. Quant Imaging Med Surg. 2019;9:1147–1162. 31367569 10.21037/qims.2019.04.18 PMC6629553

[8] Duong PT , Neill R , Zygmont M . Trust but verify: online management tool improves compliance and documentation of CT Quality control activities. J Am Coll Radiol. 2018;15:762–766. 29275916 10.1016/j.jacr.2017.11.011

[9] Goode AR , Snyder C , Snyder A , Collins P , DeLorenzo M , Lin PJ . Signal and contrast to noise ratio evaluation of fluoroscopic loops for interventional fluoroscope quality control. J Appl Clin Med Phys. 2019;20:172–180. 31593338 10.1002/acm2.12734 PMC6806477

[10] Ihalainen TM , Lonnroth NT , Peltonen JI , et al. MRI quality assurance using the ACR phantom in a multi‐unit imaging center. Acta Oncol. 2011;50:966–972. 21767198 10.3109/0284186X.2011.582515

[11] Firbank MJ , Harrison RM , Williams ED , Coulthard A . Quality assurance for MRI: practical experience. Br J Radiol. 2000;73:376–383. 10844863 10.1259/bjr.73.868.10844863

